# Dataset used for assessing animal and poultry production water footprint in selected countries of the MENA region

**DOI:** 10.1016/j.dib.2019.104621

**Published:** 2019-10-07

**Authors:** Hadi Jaafar, Roya Mourad, Nuhad Daghir

**Affiliations:** Department of Agriculture, Faculty of Agriculture and Food Sciences, American University of Beirut, Bliss St., Beirut, 2020-1100, Lebanon

**Keywords:** Poultry, Sheep and goats, Beef, Dairy, Bluewater, Green water

## Abstract

This article describes the data used to assess and quantify the local and imported (green, blue, and grey) fractions of unit and total water footprint of six categories of livestock animals along with their derived products in 14 selected countries of the MENA region. Interpretation of this data can be found in a research article titled “New estimates of water footprint for animal products in fifteen countries of the Middle East and North Africa (2010–2016)” [1]. These countries are Algeria, Egypt, Israel, Jordan, Kuwait, Lebanon, Mauritania, Morocco, Oman, Qatar, Saudi Arabia, Tunisia, Turkey, and Yemen. The main farm animals covered are beef cattle, dairy cows, sheep and goats, broilers, and layers. These data cover the period 2010–2016. The data show that the MENA region consumes more than 80 billion m^3^ of water every year for animal consumption, most of which is imported. The proportion of imported blue water to total imported water is higher than that of the local.

**Table:** Specifications Table

Subject area	*Water science*
More specific subject area	*Quantifying water footprint of livestock animals and their products*
Type of data	*Tables*
How data was acquired	FAOSTAT database, Water Stat- water footprint network, and literature
Data format	Raw and analyzed
Experimental factors	*Drinking water requirements, feed water footprint, origin of feed, feed mixing requirements, imports, the water footprint of animal feed at the origin*
Experimental features	*The collected input data (imports, domestic production, feed composition, feed volume, feed conversion, others) for 2010–2016 are presented and used to calculate the water footprint of livestock animals and their primary products including Beef, cow, sheep, goat, broilers, and laying hens in the 14 selected countries of the MENA region listed in the data source location.*
Data source location	*Algeria, Egypt, Israel, Jordan, Kuwait, Lebanon, Mauritania, Morocco, Oman, Qatar, Saudi Arabia, Tunisia, Turkey, and Yemen*
Data accessibility	*Data is available in the Appendix*
Related research article	New estimates of water footprint for animal products in fifteen countries of the Middle East and North Africa (2010–2016) [1]

**Table:** 

**Value of the Data** • The data are essential for understanding the total water demand associated with livestock production in the MENA region • The data help in identifying the type of animal product contributing to the most significant proportion of water use in livestock production • The data help identify local and imported virtual water components of various animal products • The data are valuable to the governments and policymakers for initiating a water management strategy that focuses on optimizing green, blue and greywater use in livestock farming, especially in countries with scarce water resources.

## 1 Data

**Fig. 1:**
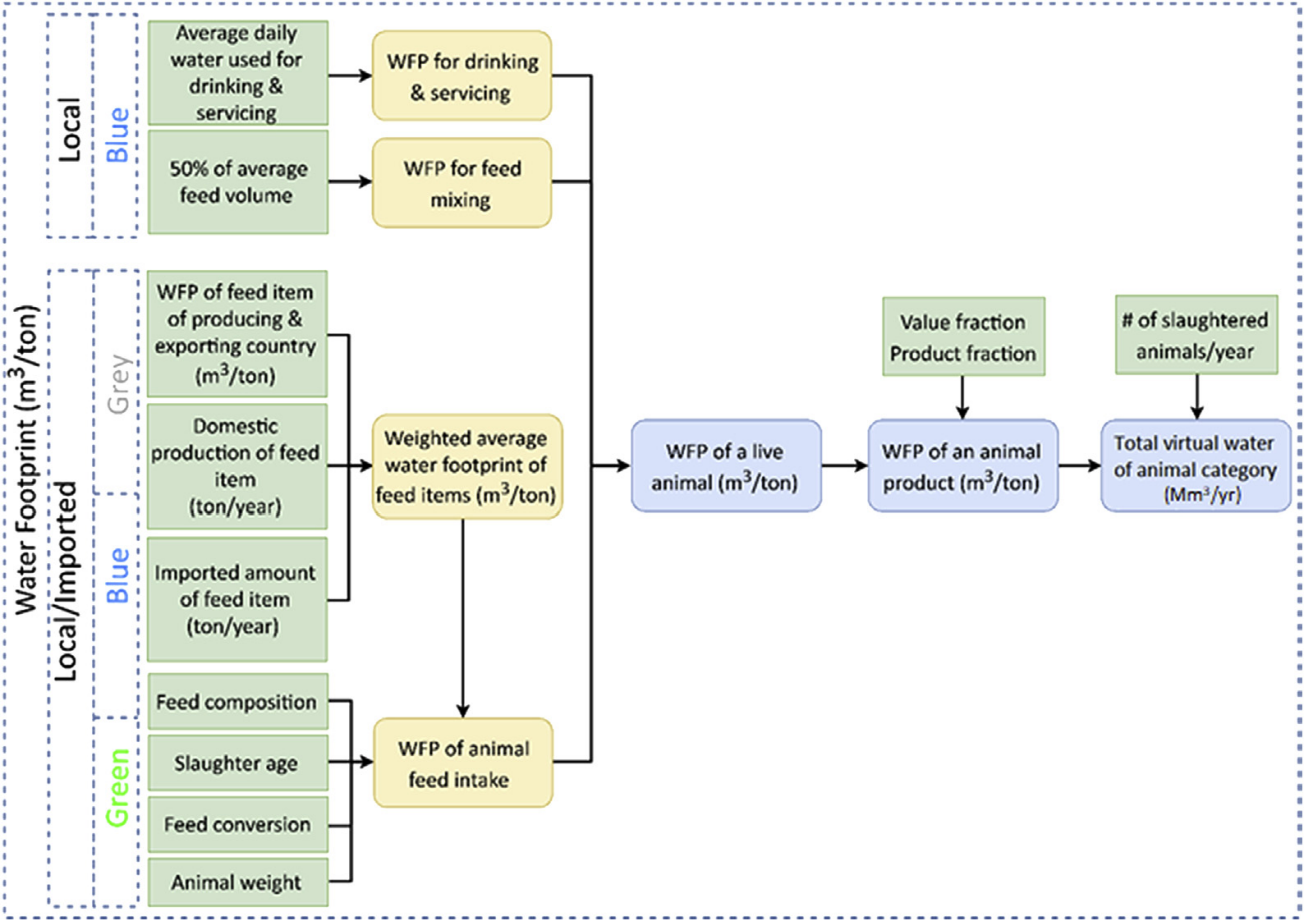
Steps in the calculations of WFP of each live animal category and animal product WFP (m^3^/ton), and total virtual water of an animal in Mega cubic metre per year (Mm^3^/yr).

**Fig. 2:**
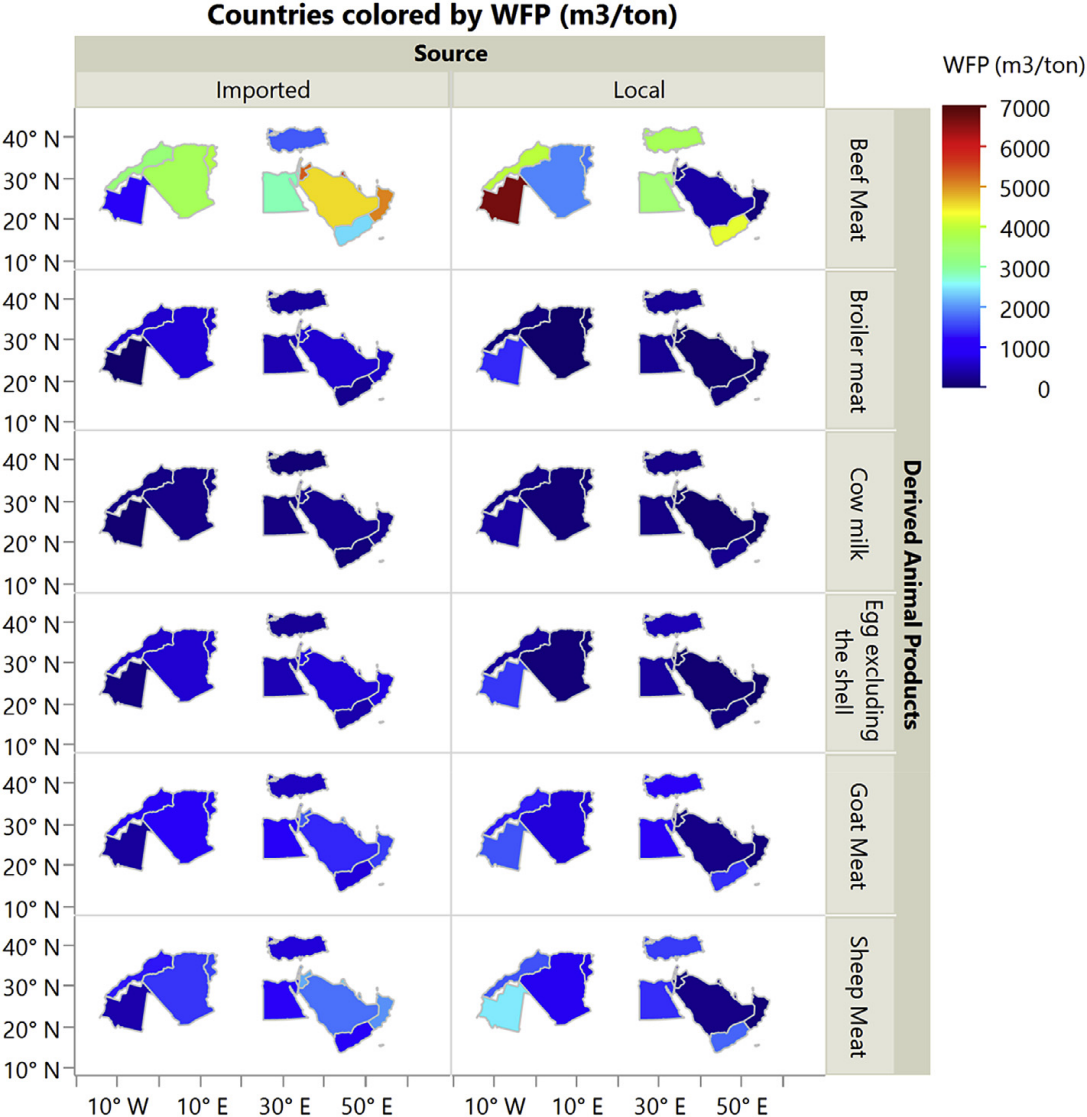
Water footprint of the animal products disaggregated into local and imported fractions for the selected countries of the MENA region.

**Fig. 3:**
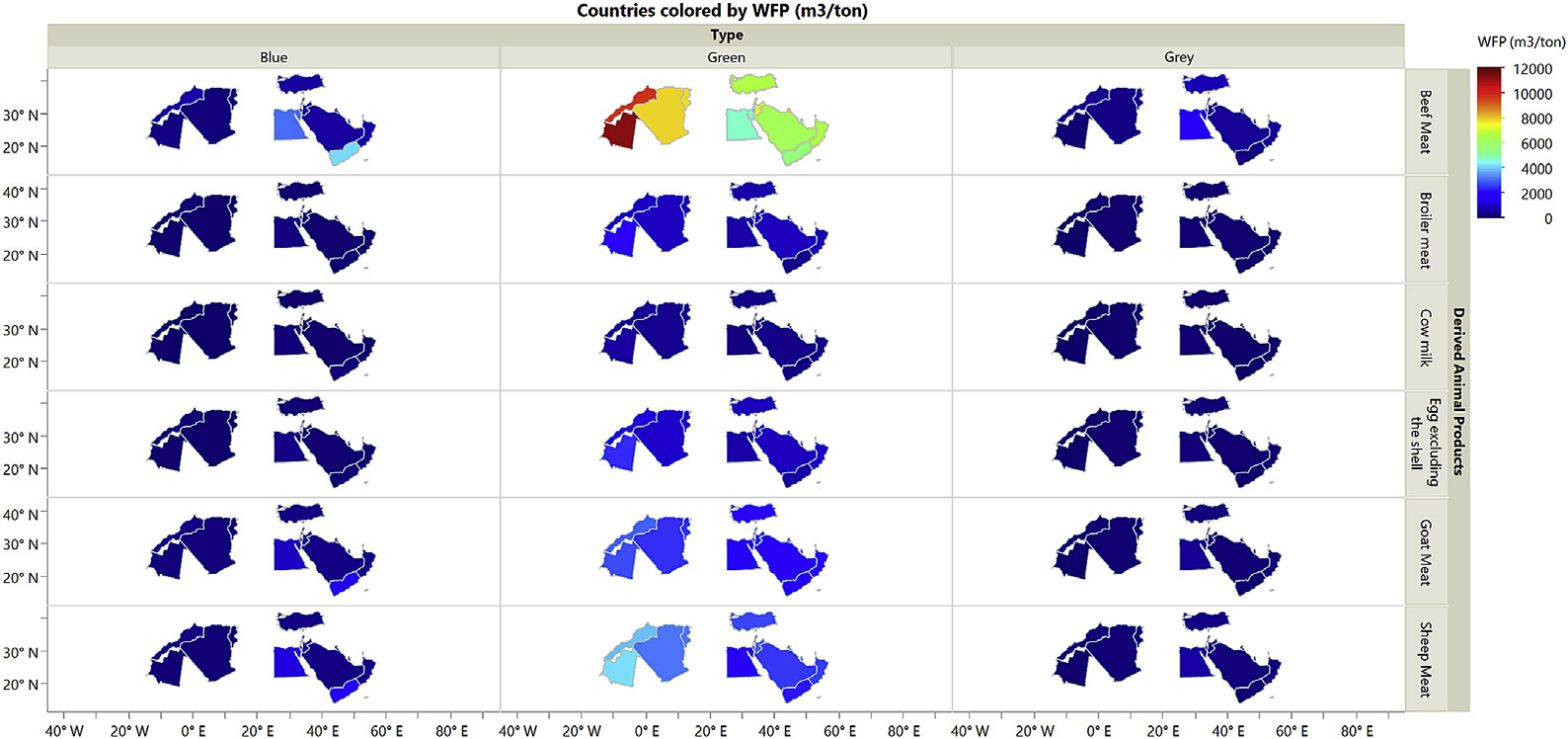
Water footprint of an animal product disaggregated green blue and grey fractions for the selected countries of the MENA region.

**Fig. 4:**
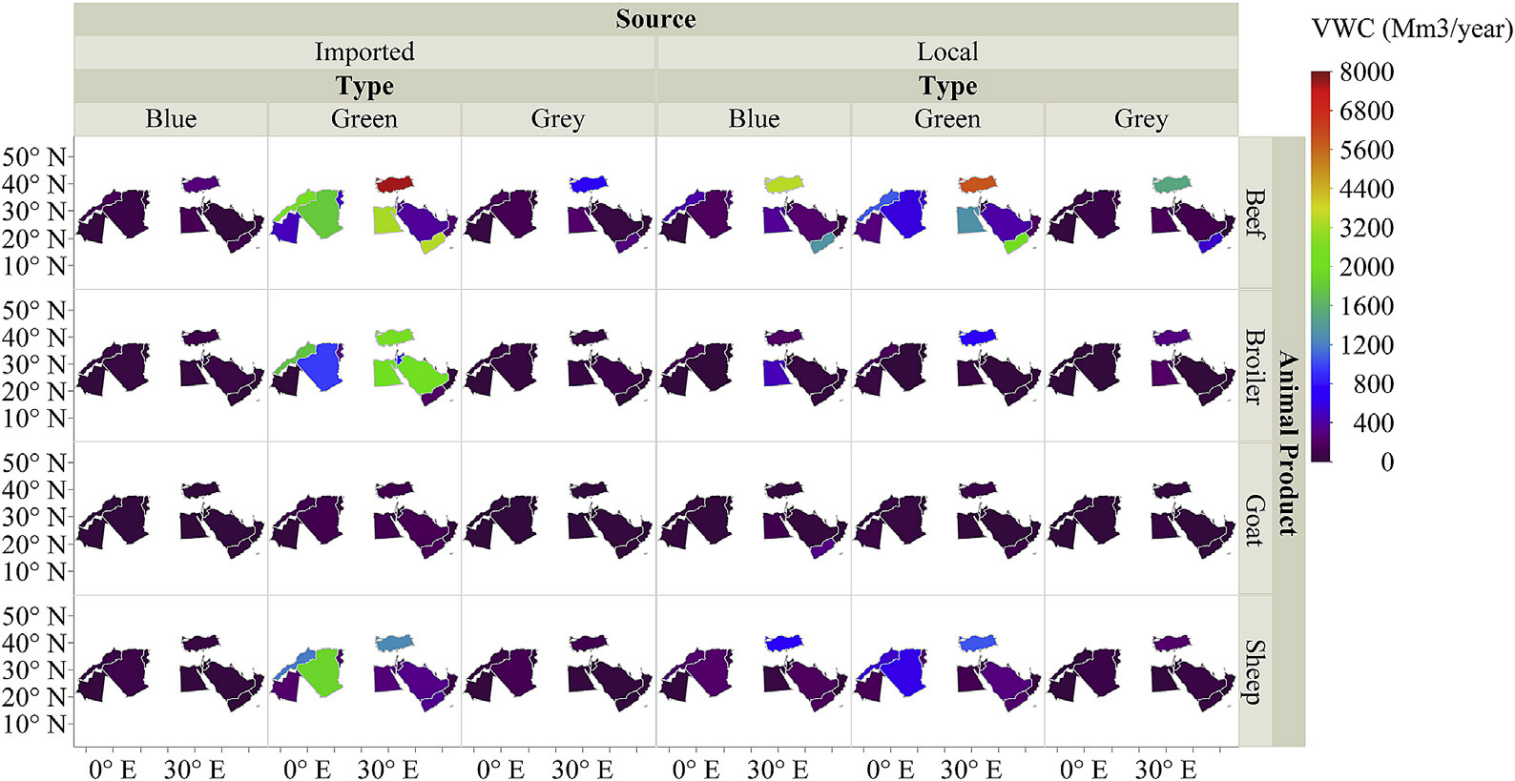
Total virtual water content for each livestock animal category disaggregated into local and imported fractions (green, blue, and grey) for the selected countries of the MENA region.

The dataset in this article describes the new water footprint for animal products in fifteen countries of the Middle East and North Africa. Fig. 1 presents the methodology for calculating the local and imported green, blue, and grey water footprint of animal products. Fig. 2 shows the weighted average water footprint (WF) of feed items disaggregated by source (local and imported fractions), and by type (green, blue, and greywater). Fig. 3 shows the WF of an animal product for the studied countries of the MENA region subdivided into green, blue, and greywater. Fig. 4 shows the values of the total virtual water content for each livestock animal category. All raw data for this work is provided as supplementary material. Table A1 shows the calculated weighted average water footprint of each feed ingredient. Table A2 represents the local and imported (green, blue, and grey) weighted average WFP of feed ingredients. Table A3 shows the WF of feed ingredients disaggregated into green, blue, and grey. The results of the sample calculations of feed WF are shown in Table A4. Table A5 presents the overall water footprint for the various animal categories (m^3^/animal). Table A6 shows the unit WF of each category animal subdivided by source (local & imported) and by type (green, blue, and grey). Table A7 provides data on the value and product fraction. Table A8 represents the WFP of animal products disaggregated into local and imported fractions for the selected countries of the MENA region. Table A9 shows the WFP of animal products disaggregated into green, blue & grey for the selected countries of the MENA region.

## 2 Experimental design, materials, and method

### 2.1 Overview

The water footprint of an animal category (WFP _animal_) in a specific country is related to feed, drinking water, and service water consumption. This WFP is usually categorized into three components: blue, green, and grey. In the definition of these components, we follow those described in Ref. [2]. The term blue water is used to refer to water originating from surface sources or groundwater sources (i.e., diverted or pumped), while greywater is the amount of water needed to assimilate the load of pollutants produced by the production system. The term green water is used to describe water received directly from rainfall and stored in the crop active root zone. In this study, the three components are further distinguished by source local and imported (i.e., whether the consumption is local to the country or imported from outside the MENA region). Fig. 1 shows the methodology for calculating the local and imported green, blue, and grey water footprint and virtual water of animal products.

### 2.2 Data acquisition

To calculate the WFP of feed, we first calculated the weighted average water footprint. The input data to the weighted average water footprint model are data on the production and imported quantities of feed items specific to each country. These data were downloaded from the FAOSTAT database [3]. The database provides statistics on 173 crops.

Another parameter used as an input to the weighted average water footprint model is the water footprint of feed item for domestic production and that of the exporting countries. The water footprint of feed is defined as the specific water demand per crop in the country where the crop is produced, divided into green, blue and grey. These data were downloaded from WaterStat-water footprint network [4]. This database provides the green, blue, and grey water footprint of crops.

Other input data include the average weight, slaughter age, feed conversion (the efficiency of which the animal body can convert the feed into the desired product) of each animal obtained from Refs. [[5], [6], [7], [8], [9], [10]], and percentage feed composition obtained from Refs. [6,7,[11], [12], [13]]. These data were used as input parameters for the WFP of feed calculations. Data needed to calculate the water footprint from drinking and servicing include the average daily water requirements for both drinking and servicing of each category animal. The average feed volume is used as input to get the water footprint originating from feed mixing as it is 50% of the average feed volume.

To calculate the WFP of an animal product, the WFP of a live animal is multiplied by the value fraction and then divided by the product fraction. The product fraction is the amount of the primary product (in ton) obtained per ton of a live animal. The value fraction is the market value of the primary product of an animal divided by the sum of the market value of all products of that corresponding animal. These data were obtained from Lebanese farms and were validated through data obtained from the literature [2,14]. Data on the average number of livestock animal slaughtered in a year were downloaded from FOASTAT [3], and they were used as an input for the calculation of the total WFP of each livestock animal expressed in Mega cubic metre per year (Mm^3^/yr).

### 2.3 Weighted average water footprint of feed item (WFP’)

#### 2.3.1 Local WFP’ (green, blue, and grey)

To get the local fraction of the weighted average water footprint of a feed item, we multiply this production by the water footprint of that ingredient and divide it by the total (imports and production).

#### 2.3.2 Imported WFP’ (green, blue, and grey)

For the imported fraction, we multiply the imported amount of the feed item by its corresponding water footprint in the exporting country. The result of this multiplication was summed and then divided over the total (imports and production).

### 2.4 Water footprint for the feed of a livestock animal (WFP_feed_)

WFP_feed_ per year was disaggregated into local and imported (green, blue, and grey) (Table A4) as per the below:

#### 2.4.1 Local WFP_feed_ (green, blue, & grey)

The average feed volume (AFV) of each feed ingredient was calculated for each animal in Lebanon. AFV was multiplied by the local WFP’ (green, blue, and grey). The result of this multiplication (represented as TWFP Local in Table A4) was then summed to get the total local water for feed per year.

#### 2.4.2 Imported WFP_feed_ (green, blue, & grey)

AFV was multiplied by the Imported WFP’ (green, blue, grey). The result of this multiplication (represented as TWFP imported in Table A4 was then summed to get the total imported water for feed per year.

### 2.5 Overall water footprint of a livestock animal (m^3^/animal)

#### 2.5.1 Water footprint of feed intake per animal (WFPFT)

In order to obtain the water footprint of feed intake per animal (WFPFT), we multiply the total water for feed obtained from Table A4 by the slaughter age of each animal.

#### 2.5.2 Water footprint of drinking & servicing per animal (WFP _drink_ & WFP _service_)

To get the total drinking (WFP _drink_) and servicing (WFP _service_) demand for each animal (m^3^/animal), we multiply the amount of water used for drinking and servicing in a year by the slaughter age of each animal.

#### 2.5.3 Water footprint for total feed mixing per animal (WFPMT)

To get the WFP for the total feed mixing (WFPMT) (m^3^/animal), we multiply 50% of average feed volume by the slaughter age of each animal.

#### 2.5.4 Local & imported (green, blue, & grey) overall water footprint of a livestock animal (m^3^/animal)

The above results on WFPFT were disaggregated into local & imported (green, blue, and grey) and that of WFP_drink_ & WFP_service_, & WFPMT were assumed to be local and blue for the easiness of calculations. Successively, WFPFT, WFP_drink_ & WFP_service_, & WFPMT are summed to get the overall water footprint for category *i* animal (m^3^/animal) (WFPi). These calculations are presented in detail in Table A5.

### 2.6 Local & imported (green, blue, & grey) unit WFP of each category animal (m^3^/ton)

Consequently, the obtained results of overall local & imported WFP (green, blue, and grey) from Table A5 were then divided by the live weight of each animal category in order to get the unit WFP of each category animal. The results of this division are shown in Table A6 subdivided by source (local & imported) and by type (green, blue, and grey).

### 2.7 Local and imported (green, blue, & grey) WFP of livestock product (m^3^/ton)

The results on the unit WFP of *i* category were multiplied by the value fraction and then divided by the product fraction to get the water footprint of each animal product (Table A7) subdivided by source (local and imported) and by type (green, blue, and grey).

### 2.8 Total WFP or virtual water content for each livestock animal category

To calculate the total WFP for each livestock animal category, we multiply the WFP of the animal product obtained from Table A7, by the weight of the livestock animal and by the number of the slaughtered animal in a year. The average number of slaughtered animals for the period 2010–2017 for each country is presented in Table A10 as an input to the preceding multiplication. The results are also subdivided by source (local and imported) and by type (green, blue, and grey). The values of the total virtual water content for each livestock animal category are shown in Fig. 4.
